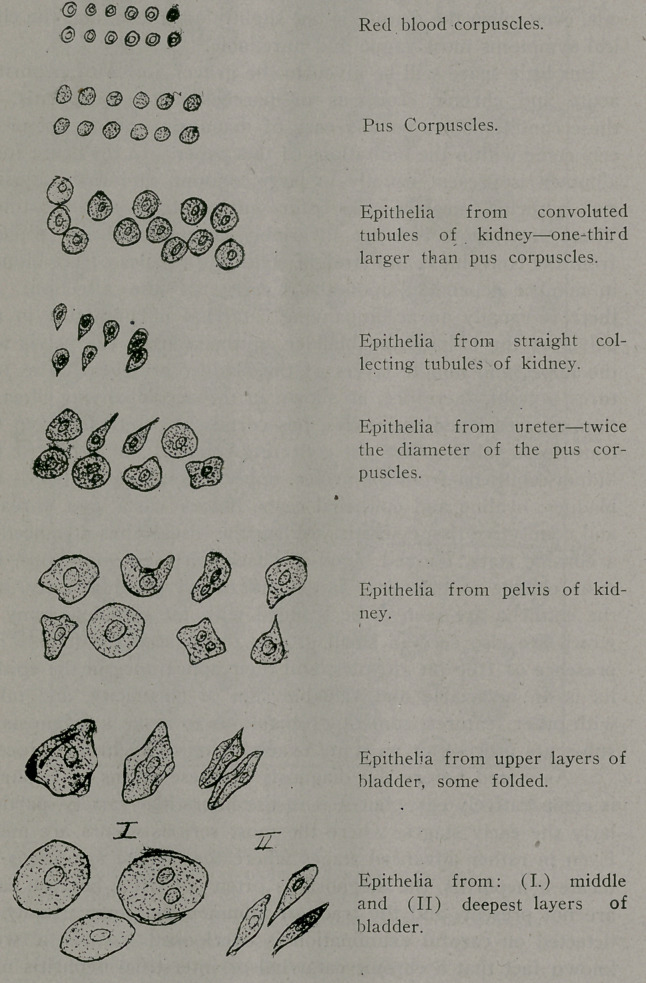# The Diagnosis of Incipient Pathological Changes in the Kidney by Means of Renal Epithelium

**Published:** 1908-03

**Authors:** A. T. Gaillard

**Affiliations:** Atlanta


### THE DIAGNOSIS OF INCIPIENT PATHOLOGICAL CHANGES IN THE KIDNEY BY MEANS OF RENAL EPITHELIUM.*

BY A. T. GAILLARD, M. D., ATLANTA.

In most of the published works on urinalysis, the subject of epithelia found in the urine receives but poor and most unscientific attention. It is usually discussed only slightly, commented upon vaguely, and then dismissed as unworthy of serious attention.

The reasons for this neglect of a highly important aid to a correct microscopical diagnosis of Genito-Urinary lesions are two-fold—1st, many authors are unwilling to devote the necessary time and research to the examination, comparison, and tabulation of thousands of specimens, and sometimes give the most positive adverse opinions on a subject which they have not taken the trouble to investigate, except in a most imperfect manner. 2nd. other authors unfortunately accept without question the dicta of previous investigators, and with only slight changes in phraseology, record them as the result of their own work.

It remained for Carl Heitzmann to point out and demonstrate conclusively that the various epithelia found in the urine may be differentiated and respectively assigned to the mucous membranes from which they were shed. This work has been carried on and perfected in the most scientific manner by his son, Louis Heitzmann, and the wonderful results he has obtained, the absolute fidelity with which his microscopical findings tally with the clinical picture of the case under observation, can be attested by hundreds of his students all over the country.

It has been the writer's privilege to see much of this work, to see diagnoses made of Genito-Urinary lesions when the clinical history was entirely lacking, to see them confirmed when this history was finally obtained, and to work out himself (very poorly it is true) some of these results in Dr. Heitzmann's laboratory.

After these remarks it is hardly necessary to state that no claim to originality is made for this paper; it is sufficient satisfaction to be allowed an opportunity to emphasize in the strongest

* Read before Fulton County Medical Society Feb. 2[illegible]th, 1908.

Acute Catarrhal Pyelo-Nephritis and Cystitis (after Heitzmann), X 500.

R B.—Red blood-corpuscles.

P C.—Pus corpuscles.

C E.—Epithelia from convoluted tubules of kidneys.

U E.—Epithelia from ureter.

U B.—Epithelia from upper layers of bladder.

M B.—Epithelium from middle layer of bladder.

P E.—Epithelia from pelvis of kidney.

U A.—Uric acid.

U G.—Uric acid gravel.

O C.—Calcium oxalate.

U S.—Sodium urate.

possible manner the great value of these investigations as an aid to diagnosis in all patholölgical conditions of the Genito-Urinary tract, especially those affecting the kidney, which will be discussed in this paper.

Before proceeding to describe the epithelia, having their origin in the kidney, a few words should be said about the varieties of epithelia found in the body. These are flat or squamous, cuboidal or oval, and cylindrical or columnar. They may occur either in a single or in several layers, when the latter, the flat compose the upper, the cuboidal the middle, and the columnar the deepest layer.

A simple epithelial lining is found in the uriniferous tubules of the kidney, and the stratified variety occurs in the pelvis of the kidney, and the ureter.

Those authorities who claim that the various epithelia cannot be differentiated have at least been compelled to modify their views since the advent of ureteral catheterization. One author states most positively that those from the bladder cannot be differentiated from those having their origin in the pelvis of the kidney. He is now able to see the errors of his ways by a comparative study of specimens obtained in the ordinary way, and by the ureteral catheter. In the writer's opinion, it is only a question of time before all authorities will agree, even if grudgingly, that most epithelia seen in the urine may be accurately assigned to the various mucus membranes of which they were originally a part.

It may now be stated without much fear of contradiction that the presence of epithelia in urine, with the exception only of the squamous from the bladder, and from the vagina in the female, invariably indicate a pathological process of some kind. This morbid process is usually an inflammatory one, the seat of the lesion being determined by the variety of epithelia predominating, and its severity by the number present in a given case.

As I have limited myself in this paper to a discussion of the early pathological changes taking place in the kidney, the accompanying illustrations show only the epithelia coming from the kidney and its pelvis, also the ureter and bladder, the latter being usually present in such inflammations.

It will be seen at a glance that the important diagnostic point lies in the comparative sizes of the epithelia, and for this the pus the corpuscle is taken as the standard. Pus corpuscles are present in every inflammation, and while varying in size in different individuals, vary but little, if at all, in a given case. When small cuboidal bodies are seen, distinctly larger than the pus corpuscle, and usually containing a nucleus, these are epithelia from the convoluted tubules of the kidney. Their relative size never varies, in diameter they are one-third larger than the pus corpuscle, and when seen in the urine a nephritis is present, even though repeated examinations fail to reveal the presence of casts. Just here the common error of relying upon casts before making a diagnosis of nephritis should be pointed out and condemned, for this disease may exist for years in a mild form without the presence of casts, and even in the more intense varieties of chronic interstitial nephritis casts are very seldom found.

Of rarer occurrence, and when present in moderate or large numbers, denoting a more intense inflammation, are small columnar epithelia from the straight collecting tubules of the kidney. In the severer varieties of nephritis these may be present in large numbers, they are frequently seen filling an epithelial cast, and cannot be mistaken for any other epithelia originating in the Genito-Urinary tract.

Epithelia from the ureter are usually round, though sometimes irregular, and are invariably about twice the diameter of the pus corpuscle. The columnar variety, coming from the deeper layers, is sometimes present when the inflammation is more intense, or due to mechanical injury, such as impacted calculus,, or the traumatism sometimes inflicted by the ureteral catheter.

Epithelia from the pelvis of the kidney are lenticular or pear-shaped, or cuboidal, except those from the deeper layer, which are columnar in shape. They are distinctly smaller than those epithelia derived from the bladder, and larger than those from the ureter.

Epithelia from the bladder consist of three distinct layers, flat or squamous from the upper layer, which variety may be seen in normal urine, and when unaccompanied by other epithelia, are of no significance; cuboidal, which always indicate some degree of injury to the mucus membrane; and columnar, which when present are the result of some deep-seated injury, such as ulceration, hemorrhage, tumor of the bladder, etc.

If a careful study is made of the epithelia mentioned and their comparative sizes accurately gauged with a magnifying power of about 450 diameters, it will be impossible to err. As

Chronic Catarrhal Pyelo-Nephritis and Cystitis (after Heitzmann) X 500.

R B.—Individual red blood globule.

*C E.—Epithelia from convoluted tubules of kidney.

*P E.—Epithelia from pelvis of kidney.

S E.—Epithelia from straight collecting tubules of kidney.

*U E.—Epithelia from ureter.

*M B.—Epithelia from middle layers of bladder.

*P C.—Pus corpuscles.

F G.—Free fat globules.

*All containing fat globules showing chronicity.

the practical application to all that has been said, I shall endeavor to show that a correct diagnosis may be reached after these methods, even when the disease is but slightly advanced, and the clinical symptoms most vague and unreliable.

But little space will be given to the graver forms of nephritis, acute and chronic croupous or parenchymatous nephritis, as these conditions are usually easy of diagnosis, and do not properly come within the limitations of this paper. In the acute form albumen is present usually in large amount, and casts, hyaline and epithelial, must be seen before such a diagnosis is justified. These casts, however, are invariably accompanied by epithelia from the convoluted and straight collecting tubules of the kidney, in number dependent upon the severity of the affection. As there is usually an accompanying catarrhal inflammation in the pelvis of the kidney and bladder, epithelia from the pelvis and the upper and middle layers of the bladder are seen. The features present, therefore, as shown in the accompanying illustration, are red blood-corpuscles, pus corpuscles, epithelia from the convoluted tubules, straight collecting tubules, and pelvis of the kidney, epithelia from the ureter, upper and middle layers of the bladder, hyaline and epithelial casts, mucus casts and threads, and connective tissue shreds. When the disease has advanced to a chronic state, the red blood-corpuscles are less numerous, the casts change to granular, fatty, and mixed granular-fatty, and the epithelia are seen to be studded with fat globules, many of which are also seen in small groups throughout the field. The presence of free fat globules, and their appearance in the epithelia is an invariable and valuable sign of chronicity, and taken with other features, sometimes enable us to make a diagnosis of sub-acute process, or an acute exacerbation of a chronic process.

As stated before the diagnosis of these forms of nephritis is comparatively easy, but it is in the interstitial variety, particularly the early stages, where the most serious errors are made. Even in rather advanced stages where the clinical symptoms indicate a nephritis, the diagnosis is often not made because casts are not present, and the trace of albumen that may usually be detected on careful examination is overlooked. It is a well-known fact that a chronic catarrhal or interstitial nephritis may exist for years without manifesting itself by any but the vaguest of clinical symptoms. It is equally true that albumen may be very often absent, or give the reaction of only a slight trace, and

Diagram showing comparative sizes of corpuscles and epithelia (X 500).

Red blood corpuscles.

Pus Corpuscles.

Epithelia from convoluted tubules of kidney—one-third larger than pus corpuscles.

Epithelia from straight collecting tubules of kidney.

Epithelia from ureter—twice the diameter of the pus corpuscles.

Epithelia from pelvis of kidney.

Epithelia from upper layers of bladder, some folded.

Epithelia from: (I.) middle and (II) deepest layers of bladder.

even a true cirrhosis of the kidney may exist without the presence of casts. But how often are these facts overlooked? How often do we see the diagnosis of nephritis abandoned because repeated examinations fail to reveal casts. And if the trace of albumen is detected, how often is refuge sought in the vague appellation of functional, physiological, or postural albuminuria. For my part I fail to see why any importance whatever should be attached to this postural or orthostatic albuminuria. It is natural enough, when the pathological change taking place in the kidney is in its incipiency, for albumen to be present when the organ is comparatively speaking at rest, and for it to appear during the periods of digestion and active exercise. These are the cases where a careful examination will show that an inflammatory process is going on, evidenced by a moderate number of red blood-corpuscles, pus corpuscles, and epithelia from the convoluted and straight collecting tubules of the kidney. When these features are present the diagnosis of a catarrhal nephritis can be made, and if not made and treatment instituted to remove the cause, the inflammatory condition will be augmented, and sooner or later the unfortunate patient will experience all the graver clinical symptoms that assuredly might have been avoided had an early diagnosis been made.

Exception must be made, however, to the adolescent albuminuria, sometimes observed in athletics after very severe or prolonged physical exertion. Under such conditions, the kidney being over worked, just as the heart and other organs, resents the strain and albumen in the urine follows, though microscopical examination reveals no organic lesion. If the tax upon its eliminating powers be long continued, such organic lesion will in time undoubtedly develop, just as the additional strain put upon the heart results in cardiac hypertrophy.

When, as frequently happens, the inflammation has extended to the pelvis of the kidney, the characteristic cuboidal and pear-shaped epithelia are seen, and justifies the diagnosis of a pyelonephritis. There are usually present, also, epithelia from the ureter and the upper and middle layers of the bladder.

The acute catarrhal process just described, in a great many cases becomes a chronic one, the result usually of a failure to recognize the condition by a careful microscopical examination of the urine after the methods indicated. When chronic, the features change in the following manner: Red blood-corpuscles are either absent, or if present, occur in very small numbers, free fat globules, sometimes in groups, are scattered throughout the field, and appear in varying numbers within the pus corpuscles and epithelia. When present in large numbers they indicate a beginning fatty degeneration of the kidney. As in a nephritis of the croupous or parenchymatous variety, a sub-acute process may be diagnosed by the appearance of both red blood-corpuscles and fat globules, the former in moderate and the latter in small numbers.

When the features described in a typical case of catarrhal nephritis are present only to a slight degree, a trace of albumen or none at all, very few red blood-corpuscles, pus corpuscles, and epithelia from the convoluted tubules of the kidney, the condition is not severe enough to warrant a diagnosis of nephritis, and the diagnosis of congestion or hyperemia of the kidney may be made. This is frequently to be seen as an accompaniment to other diseases, it may be the result of simple exposure, or the ingestion of certain drugs, as copaiba, turpentine, etc. It is frequently seen in cases of prostatitis of gonorrheal origin, and may also be due to the passage of large quantities of salts, such as uric acid and calcium oxalate. It is highly important that an early diagnosis be made in these cases, for the cause is easily removed, but if not eliminated by prompt treatment a true inflammation with its resulting nephritis will sooner or later manifest itself.

Before closing mention should be made of a very valuable aid at our command in the prognosis of all diseases for which the urine is examined microscopically. It was first announced by Carl Heitzmann in 1879 that the constitution of an individual may be accurately determined by the appearance of the pus corpuscle, and thousands of examinations have proved beyond question that his assertion is a correct one, and is always borne out by the clinical evidence of the case. When the pus corpuscles appear as coarsely granular, highly refractive bodies, it is a sure indication that the constitution is an excellent one. This coarse granulation is due to a large amount of living matter, and the less the amount of living matter, the finer the granulation and the poorer the constitution. A good constitution is evidenced by somewhat less coarse granulation and less refraction. When the constitution is a poor one the pus corpuscles are very finely granular, of little refraction, one or more nuclei are seen, and the edges are frequently irregular and ragged. This reasoning may be followed through all the grades of inflammation. For instance, if many coarsely granular highly refractive pus corpuscles are seen in a given case, with a moderate number of finely granular nucleated ones, the constitution was originally excellent but is now impaired by disease, the extent of impairment being dependent upon the relative ratio between the coarsely granular and the finely granular. As before stated, many studies have been made of given cases, following them from the incipiency of the disease to its fatal termination, and the result is always the same, an accurate estimate of the patient's constitution at the time of the examination.

In conclusion I must apologize for this presentation of methods requiring perhaps too much time for the general practitioner, but if I have succeeded in interesting those who devote special study to the microscopical examination of the urine, this paper will not be wholly without its justification.

407 *English-American Bldg.*

DISCUSSION.

*Dr. E. G. Ballenger* enjoyed the well-written and interesting paper by Dr. Gaillard, and has been taking considerable interest in this subject since having had a few personal conversations with the essayist upon this matter, but Dr. Ballenger said that his experience with these cells was too limited to discuss their significance in an intelligent manner.

He had noted one characteristic of prostatic cells, and that is that they are always more or less degenerated and do not stain clearly when the gland is inflamed; this is due to the digestive action of leucoprotease, a ferment liberated from the leucocytes when they are in a faintly alkaline medium. It is by this substance that the proteids secreted in the prostatic follicles are changed into albumose, which is constantly present in prostatitis.

I am very glad to have had the privilege of hearing Dr. Gaillard's paper, and feel that if we could recognize these cells accurately we would have an adjunct of value to add to our present methods of diagnosis.

*By D. H. F. Harris.* I have listened with much interest to the paper of Dr. Gaillard, and much regret that I feel constrained, in the interest of scientific accuracy, to disagree with pretty nearly everything that he has said. The questions that he has raised are by no means new, and have been discussed by microscopists in the past. I recall distinctly having heard a paper on the subject read by Dr. Longstreath before the Philadelphia Pathological Society some eighteen years ago. I also remember that it was the universal concensus of opinion of those present—and they were the best microscopists in Philadelphia—that nothing of diagnostic value could be determined from the character of the epithelial cells found in the urine.

I would particularly take exception to the statement that it is possible to determine the origin of epithelial cells by their shape or size, or by a combination of the two. It is well known that the sizes of epithelial cells of the different tubules of the kidneys vary greatly; and I have no hesitation in saying that they in no way differ from the cells that are found in the deeper layers of the pelvis of the kidney or ureter, and even in the bladder itself. It is known that the double layer of epithelial cells that form the inner covering of the bladder vary greatly, the form depending upon the state of construction of this viscus. If the organ be greatly contracted, the superficial cells are cubical or cylindrical, while in the dilated bladder they are flattened, and resemble very closely squamous epithelium; the cells in the deeper layer are long, irregular and string-like bodies in the former condition, and cubical in the latter. The cells covering the pelvis of the kidney and ureter in every way resemble the deeper layer of cells found in the contracted bladder.

I do not agree with the statement that casts are frequently absent in Bright's disease. I recall only two instances where I have seen patients presenting the clinical picture of Bright's in the urine of which casts were never demonstrated. In both cases the patients were suffering apparently from a very severe parenchymatous nephritis, and both died in a comparatively short period of time. The urine contained a great quantity of albumen. I have always believed that a more careful search would have revealed the presence of casts. It is my uniform experience that while casts are not plentiful in chronic interstitial nephritis, they may be always found by obtaining the sediment from a twenty-four-hour specimen of the urine, and then centrifugating it.

I do not know where the doctor gets his authority for the statement that parenchymatous nephritis is accompanied by catarrhal inflammation of the pelvis of the kidney and bladder. I have certainly never seen anything in the urine, nor have I observed evidences post-mortem that would lead me to believe that this was a common occurrence. I grant that it is possible, but believe that it is extremely rare.

I regret to have to combat the doctor's views regarding postural albuminuria. I know of instances where young men suffered from a condition of this kind as much as fifteen or eighteen years ago, and they are to-day in perfect health. In some cases where the patients formerly had this trouble I have had an opportunity of examining the urine recently, and in every instance it was absolutely normal. I do not believe that conditions of this kind are followed by serious diseases of the kidneys, and do not regard, therefore, the presence of albumen under such circumstances being of any particular significance.

I desire also to protest against the statement that the character of the pus cells found in the urine indicates the nature of the constitution of the individual in question. A division of the leukocytes into those merely containing large or small granules is hardly up to the present state of knowledge concerning these bodies. We should at least have a right to know to which group of Ehrlich's classification they belong. This assumption seems all the more remarkable in view of the fact that it is known that under different circumstances the number of leukocytes varies greatly, and that in different diseases the proportion of the various kinds is changed. We would quite conceive that a man might have a great number of leukocytes with large granules in one pathological condition, and cells of a finely granular character in some other affection.

The most pernicious feature, it strikes me, of Heitzmann's views lies in the fact that he has necessarily assumed from the clinical histories of his cases the pathological condition, and he has made his examinations of the urine with no other criterion to judge from. Could we assume always to know the morbid state without an examination of the urine, as he has done, I see no good reason for making such examinations at all. Under such circumstances it is certainly superfluous. Perhaps the clinician can view with more equanimity than a pathologist such propositions as these, but to a man who has spent a few years doing post-mortems, the assumption of any knowledge concerning the state of the internal organs from symptoms not of the most absolute kind, seems far-fetched. I protest then against the assumption on the part of Dr. Heitzmann and the writer of knowing things that only a post-mortem could reveal, and this, I take it, has been done in a very small percentage of their cases.

*By Dr. Gaillard* (Closing). It is very gratifying to me that this paper has been so freely and fully discussed, even if most of the gentlemen disagree in part or in toto with the writer. The paper was written for this especial purpose, to induce discussion, and as a plea for more accurate and painstaking work along the line of microscopical diagnosis of pathological conditions as shown in the urine.

In reply, I shall pay particular and categorical attention to the discussion of Dr. Harris, as he has seen fit to differ so widely with the exact and scientific work done by Heitzmann. I am quite sure that Dr. Harris is not aware of the extent to which the conclusions reached by Heitzmann are now accepted and followed by leading pathologists. I am quite ready to believe that some of the best microscopists in Philadelphia eighteen years ago confessed to an inability to determine anything of diagnostic value from the character of epithelial cells found in the urine, and in spite of the progress made since that time, some of these men doubtless hold the same views to-day, having dismissed the subject for all time eighteen years ago. I wish to emphasize again that Heitzmann's work is pioneer in its character, must be studied out step by step in the most painstaking manner, and most of the criticisms expressed are the result of insufficient or incomplete work, or a deliberate refusal to accept what he has clearly proved and demonstrated.

I am unable to understand why Dr. Harris should state that it is well known that the sizes of epithelial cells of the different tubules of the kidney vary so greatly, for as a matter of fact they do not so differ in the urine. He then states that they cannot be differentiated from those having their origin in the pelvis of the kidney or ureter, or even in the bladder itself; a virtual confession that the source of all epithelia is in doubt, and then he proceeds to discuss the varying appearance of epithelia from a contracted and dilated bladder. Surely this is a contradiction, for if able to differentiate these, why can he not go further and place the others where they rightly belong. But all authorities at least refer to renal epithelia, if only in a general way, and distinguish them from bladder epithelia, and if anything else is needed to refute the claim made by Dr. Harris, the ureteral catheter absolutely overturns such views as he has expressed. It will hardly be denied that epithelia found in a ureteral catheter specimen come from the kidney, and it is universally agreed that the presence of such epithelia denotes a pathological process. On the other hand such urine will never contain the epithelia generally accepted as coming from the bladder, but obtain a specimen in the ordinary way and they promptly appear, in addition to those of renal origin already mentioned. I fail to see how more positive evidence could be had.

Dr. Harris is mistaken in attributing to me the statement that casts are frequently absent in Bright's disease, particularly in the parenchymatous form. On the contrary, I went so far as to say that a diagnosis of parenchymatous nephritis could not be made unless casts were positively demonstrated, usually in large numbers, and I quite agree with him that in the two fatal cases mentioned where they were not found, they were nevertheless present, assuming, of course, that the diagnosis was correct. As regards casts in chronic interstitial nephritis, I can only say that my experience has not been the same as that of Dr. Harris, and I believe the consensus of opinion to be that casts are rare rather than common in this disease.

It is not surprising that the Doctor has failed to observe in the urine evidences of an accompanying inflammation in the pelvis of the kidney and the bladder in cases of nephritis, when he denies the presence of, or fails to differentiate the epithelia, upon which such a diagnosis is based. It is not extremely rare, but very common, and is a simple and natural result of extension of the inflammation, not more surprising than the cystitis, prostatitis, and even nephritis, that may, and do, follow chronic gonorrhea.

As regards the Doctor's views on postural albuminuria, it seems a poor argument to dismiss the condition as unworthy of notice because he has observed cases that are apparently in good health after fifteen years. I know a number of men, and Dr. Harris doubtless knows many, who have had chronic interstitial nephritis for more than fifteen years, and yet they attend to their duties as if in perfect health. My claim was, and still is, that the cause of this postural albuminuria on careful examination can be located in a functional insufficiency of the kidneys, and is evidenced by the presence of red blood-corpuscles, pus corpuscles, and renal epithelia in small numbers. It may not be a serious condition, but certainly is progressive at times.

I am really at a loss to know how to reply to the Doctor when he demands a classification of pus corpuscles according to Ehrlich. Pus corpuscles, as seen in a fresh specimen of urine, the only way they can be studied to arrive at an estimate of the patient's constitution, do not present the varied characteristics mentioned by Dr. Harris. I can only assume that he is referring to a study of stained specimens, and such a procedure is absolutely valueless for purposes of diagnosis. Pus corpuscles in fresh urine present only the characteristics I have mentioned; being coarsely granular or finely granular, highly refractive or less so, regular in outline or irregular, have one or more visible nuclei, or none at all.

The final criticism of Dr. Harris is so totally at variance with the facts, and does Heitzmann and his teachings so much injustice, that it is hard to answer, except by saying that the reverse of his criticism is true. A diagnosis is not reached by assuming the pathological condition from the clinical history; on the contrary, a correct diagnosis is often reached without the slightest aid from the clinician. It is quite true, that in order to locate the sources of the various epithelia, it was first necessary to observe carefully which ones appeared in given pathollogical conditions, but once placed upon a firm basis, this knowledge is now used for the purpose of diagnosis, independently if need be of the clinical history. Where the Doctor got his idea that the morbid state is assumed without an examination of the urine, I am at a loss to understand—certainly not from anything said in this paper. The reverse, of course, is true—the examination of the urine determines the morbid state.

If Dr. Harris wishes to limit us to the observance and detection of only those symptoms that can subsequently at the post-mortem be proved to have existed during life, and if he forbids —to use his own words—any assumption of knowledge concerning the state of the internal organs, except by the symptoms just mentioned, it seems to me that all progress would be at an end. I, for one, elect to be on the other side, and to follow as accurately as possible teaching based upon sound and scientific investigation.

## Figures and Tables

**Figure f1:**
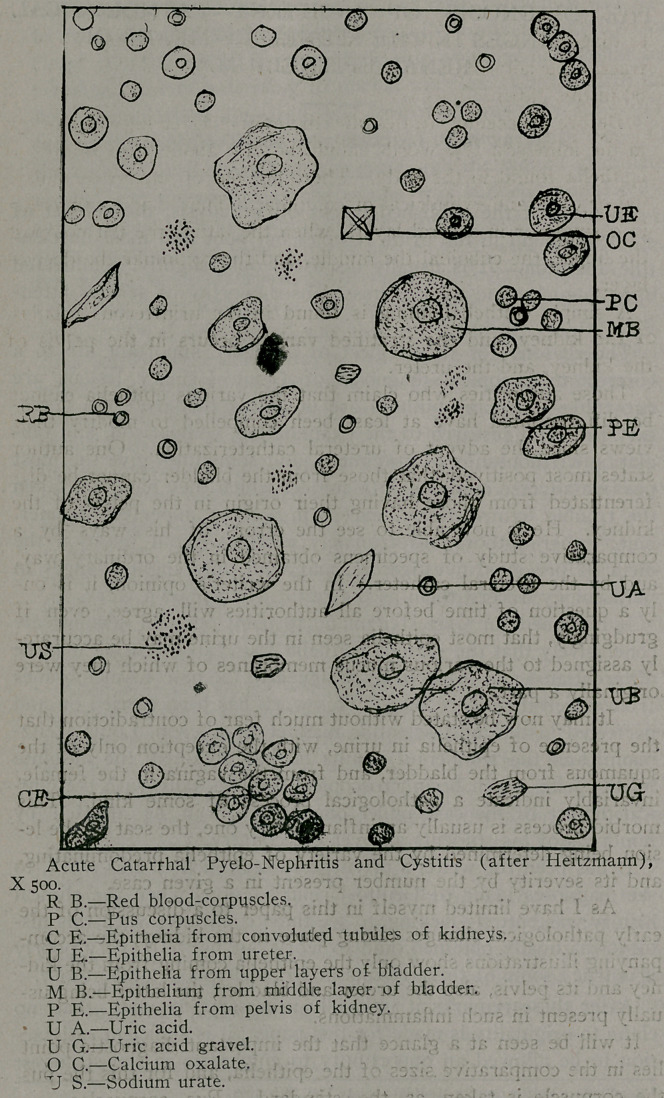


**Figure f2:**
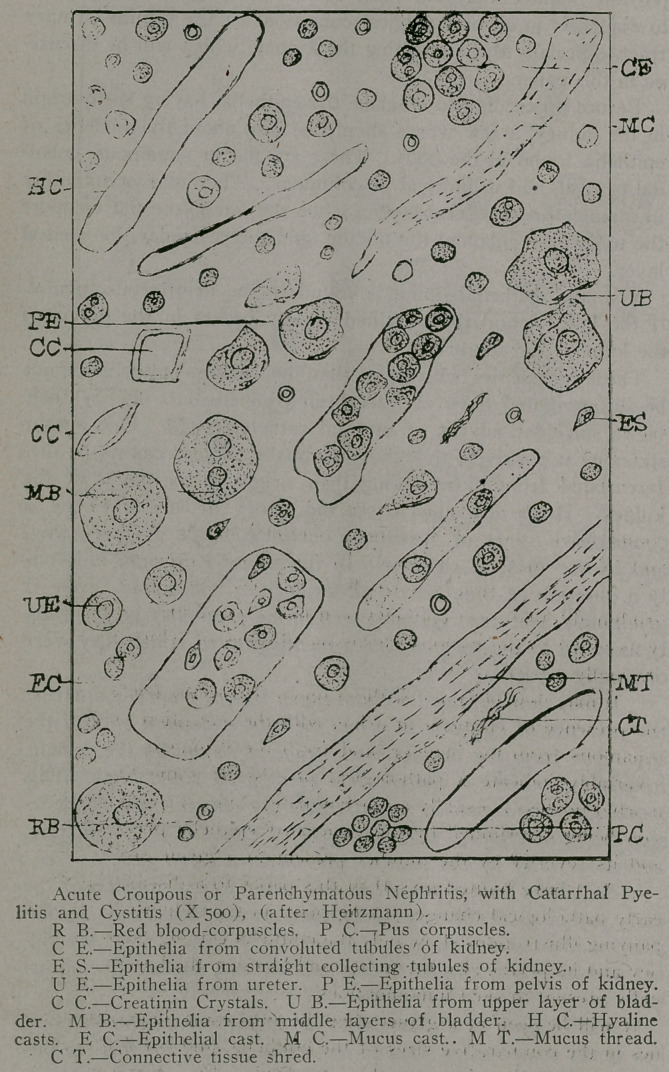


**Figure f3:**
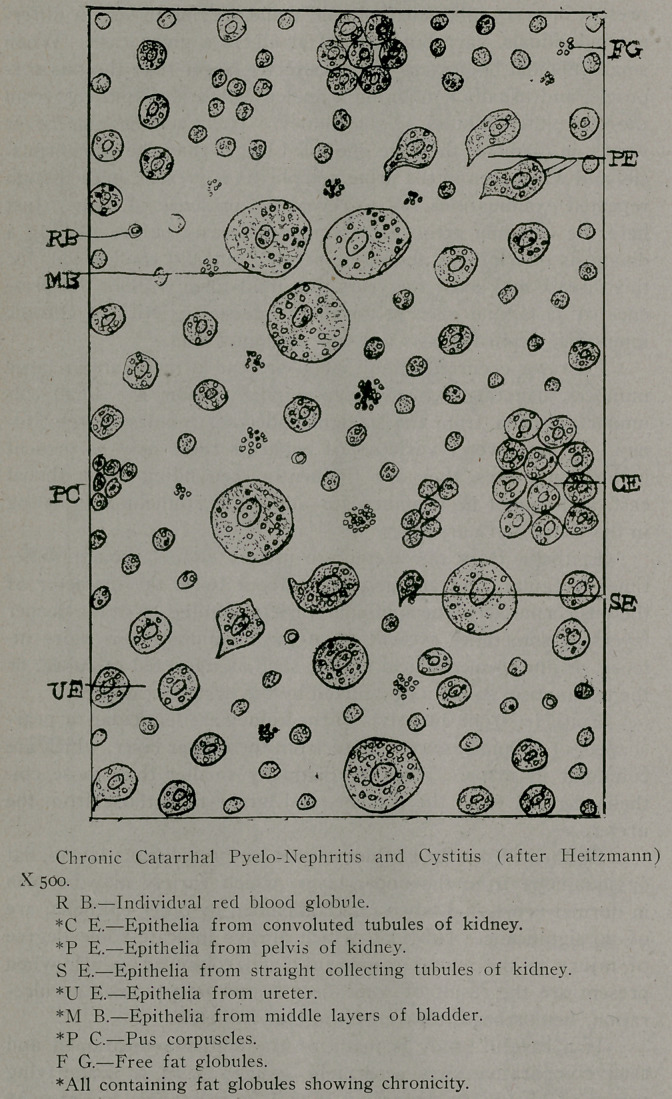


**Figure f4:**